# Calorimetric Studies of the Silver-Titanium System

**DOI:** 10.3390/molecules30193898

**Published:** 2025-09-26

**Authors:** Weronika Gozdur, Wojciech Gierlotka, Władysław Gąsior, Anna Wierzbicka-Miernik, Tomasz Czeppe, Andrzej Budziak, Agata Radziwonko, Magda Pęska, Adam Dębski

**Affiliations:** 1 Institute of Metallurgy and Materials Science, Polish Academy of Sciences, 25 Reymonta St., 30-059 Krakow, Poland; w.gozdur@imim.pl (W.G.); w.gasior@imim.pl (W.G.); a.wierzbicka@imim.pl (A.W.-M.); t.czeppe@imim.pl (T.C.); 2 Department of Materials Science and Engineering, National Dong Hwa University, Hualien 974301, Taiwan; wojtek@gms.ndhu.edu.tw; 3 Faculty of Energy and Fuels, AGH University of Krakow, Al. Mickiewicza 30, 30-059 Krakow, Poland; budziak@agh.edu.pl; 4 Department of Functional Materials and Hydrogen Technology, Military University of Technology, 2 Kaliskiego St., 00-908 Warsaw, Poland; agata.radziwonko@wat.edu.pl (A.R.); magda.peska@wat.edu.pl (M.P.)

**Keywords:** calorimetry, Ag-Ti alloys, thermodynamic properties, enthalpy of mixing, enthalpy of formation, differential scanning calorimetry (DSC)

## Abstract

Alloys from the Ag-Ti system are extremely promising and offer the possibility of versatile applications owing to their attractive properties. However, due to the experimental difficulties caused, among others, by the significant difference in melting points of the components, most of the information on the thermodynamic properties available in the literature has been obtained by computer methods. Therefore, the main aim of this work is to extend the current knowledge about the experimentally determined thermodynamic properties of selected alloys from the Ag-Ti system. Within the scope of this work, calorimetric studies were carried out using Differential Scanning Calorimetry (DSC) and high-temperature drop calorimetry measurements. The first of the aforementioned methods was used to determine the characteristic temperature of the Ag_0.43_Ti_0.57_ alloy synthesized by mechanical alloying. Using titanium hydride instead of titanium for the preparation of alloys from the Ag-Ti system has not yet been reported in the literature. This paper presents a complete structural characterization (SEM, XRD studies) of the above alloy produced by this method. The second technique was applied to ascertain the mixing enthalpy change in the alloys in the composition range between *x*_Ti_ = 0.02–0.226, and for the measurements of the formation enthalpy of the AgTi intermetallic phase. Based on the calorimetric results obtained in this study, along with the relevant thermodynamic data from the literature, the Ag-Ti phase diagram was reoptimized.

## 1. Introduction

Silver-titanium alloys are becoming increasingly important in many industrial fields, starting with applications in biomedicine as orthopedic and dental implants [1], to their use as a key ingredient in active filler alloys used in the soldering of ceramics, to metals [2]. The multitude of applications is due to their unique combination of properties, such as excellent biocompatibility, antibacterial behavior, and good mechanical strength. This system is also often part of multi-component alloys, such as Ag-Cu-Ti [2] or Ti-Mo-Ag [3], which can be used as shape memory alloys for biomedical applications. Besides these uses, Ag-Ti alloys are also being studied for their potential in protective coatings, such as Ag-Pt-TiO_2_ nanocomposite coatings [4], and Ag-TiO_2_ hybrid coatings on NiTi substrates [5].

The phase equilibria in this system have been analyzed by a number of researchers, whose results are generally in agreement with each other. The most important earlier works include [6,7,8,9,10], which were taken into account in the development of the article by Murray and Bhansali [11]. More recent work on phase equilibria in the Ag-Ti system has been developed using calculational methods based on the data available in the literature [12,13,14]. The binary Ag-Ti system is characterized by the presence of five equilibrium phases, which include the solid solutions (βTi), (αTi), (Ag), as well as the compounds AgTi_2_ and AgTi. Detailed information on their crystal structures is summarized in Table 1.

In addition, there are four invariant reactions between the phases of the system, which are: L + (βTi)→AgTi, AgTi + (βTi)→AgTi_2_, (βTi)→AgTi_2_ + (αTi), L→(Ag) + AgTi (L + AgTi→(Ag)). It should be noted that there is some disagreement in the available literature on the nature of the last-mentioned reaction. In McQuillian [8], Li et al. [13], and our previous work [14], it was described as a peritectic reaction, while Arroyave [12] and Emerenko et al. [9] describe it as eutectic. Detailed information on the temperatures and compositions of the reactions cited in the literature is summarized in Table 2. The symbols I, II, and III in Table 2 are used to denote the composition of each component involved in the listed reactions. Dezellus et al. [15] suggested the existence of a metastable miscibility gap in the silver-titanium alloy due to liquid separation that was found in the Ag-Cu-Ti ternary system. Nagase et al. [16], who observed separation in rapidly solidified melt-spun ribbons, experimentally confirmed the existence of a metastable miscibility gap.

In terms of thermodynamic properties determined experimentally, the measurement of the enthalpy of mixing of liquid Ag-Ti alloys in the concentration range between *x*_Ti_ = 0.02–0.5 using high-temperature calorimetry at 1473 ± 2 K was presented in the work of Fitzner and Kleppa [18], who show negative values of the enthalpy of mixing. The activity of titanium in Ag–Ti alloys at 1273 K were determined by Wei et al. [19], using electromotive force (EMF) measurements, and the enthalpies of formation of the AgTi and AgTi_2_ phases were measured experimentally using high temperature direct synthesis calorimetry at 1372 K by Meschel and Kleppa [20], and they amount to −1.6 (±2.4), −2.3 (±1.1) kJ/mol∙at, respectively. The calculated enthalpies of phase formation from the Ag-Ti system obtained by the CALPHAD method were presented in the work of Li et al. [13], and they agree well with the experimental results reported in [20].

Because of the limited amount of calorimetric data on the Ag-Ti system available in the literature, the primary objective of this work is to broaden the current knowledge of the thermodynamic properties of selected alloys from this system. The second goal is to evaluate the possibility of obtaining Ag-Ti alloys by mechanical alloying of titanium hydride with silver, instead of using pure metals, as proposed earlier in [21,22]. To the best of our knowledge, the use of titanium hydride as a starting material for Ag-Ti alloy preparation has not yet been reported. In this study, we present a detailed structural characterization (SEM, XRD) of the Ag_0.43_Ti_0.57_ alloy obtained by this method, as well as its DSC measurement. Finally, combining our calorimetric measurements, calculated results, and the previously reported thermodynamic data, we propose a new set of thermodynamic parameters and present the CALPHAD calculations of the Ag-Ti phase diagram.

## 2. Results and Discussion

### 2.1. Experimental Study

#### 2.1.1. Phase Analysis and Microstructural Characterization

**Figure 1:**
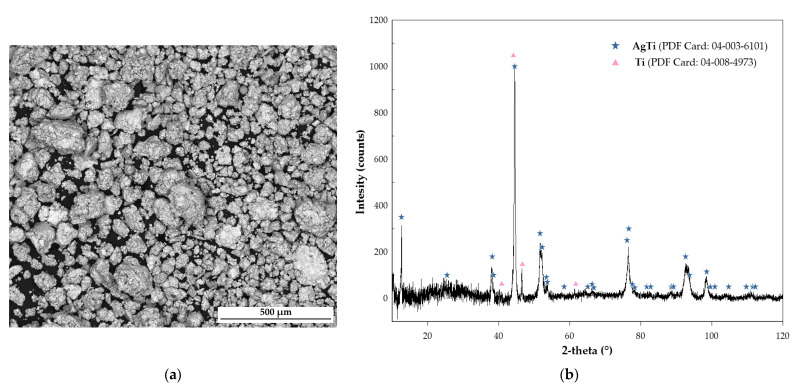
Results of structural studies of the Ag_0.43_Ti_0.57_ alloy after annealing: (**a**) SEM image, (**b**) X-ray diffraction pattern.

Samples for the Differential Scanning Calorimetry (DSC) measurement were obtained with the use of a mechanical alloying process and then analyzed with Scanning Electron Microscopy (SEM), Energy-dispersive Spectrometry (EDS), and X-ray diffraction (XRD). Figure 1a shows the SEM image of the resulting Ag_0.43_Ti_0.57_ powder, and Figure 1b shows the diffraction pattern of the powder after annealing. The average chemical composition is presented in Table 3.

#### 2.1.2. Differential Scanning Calorimetry (DSC)

**Figure 2:**
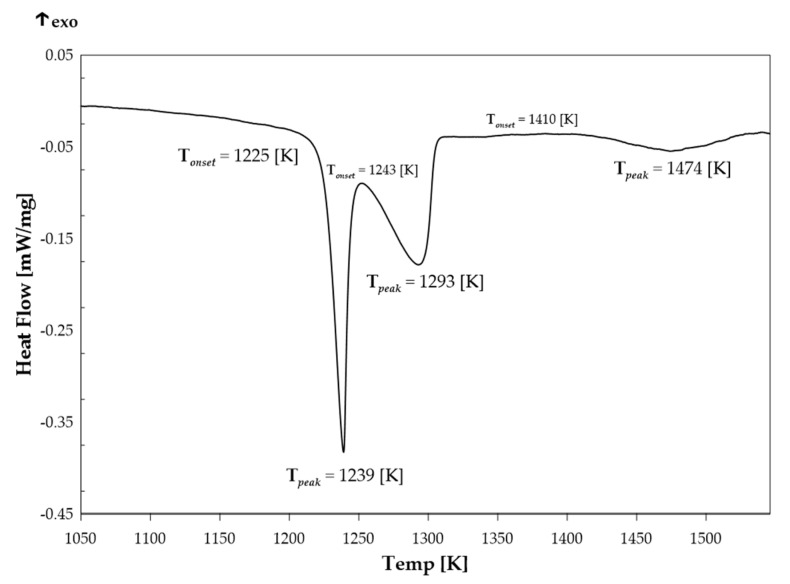
High-temperature DSC signal during heating for the Ag_0.43_Ti_0.57_ alloy.

Figure 2 shows the DSC curve of the Ag_0.43_Ti_0.57_ alloy recorded during heating of the sample. In the heating process, signals corresponding to the phase transitions in the investigated alloy are observed. The first thermal effect, consisting of two endothermic peaks, is recorded in the temperature range of 1225–1300 K, with the minimum at 1239 K and 1293 K, respectively. The onset temperature of this effect was determined using the tangent method and is estimated to be 1225 K. At higher temperatures, above 1400 K, another endothermic thermal effect is observed, with significantly lower intensity compared to the first one, with a minimum at 1474 K.

#### 2.1.3. High-Temperature Drop Calorimetry—Enthalpy of Mixing

The experimental results from two calorimetric measurements of mixing enthalpy change for selected alloys from the Ag-Ti system are summarized in Table 4 and Table 5. Detailed information on the measurement parameters for each series is summarized in Section 3.1.5 (Table 9) on Materials and Methods.

Measurements carried out with the use of high-temperature drop calorimetry showed that, within the investigated concentration range, both the integral molar mixing enthalpy change and partial molar enthalpy of Ti exhibit negative values. Despite the difficulty of performing calorimetric measurements on liquid Ag-Ti alloys (Table 4-Series A and Table 5-Series B), the obtained results can be considered consistent and repeatable. When compared with the literature data, there is some discrepancy between the reported experimental data and the calculated data. According to Fitzner and Kleppa [18], the mixing enthalpy of liquid Ag–Ti alloys is negative throughout the tested composition range, which aligns with our calorimetric measurements. Conversely, the calculated values reported in [12,13,14] indicate a positive deviation from ideality.

#### 2.1.4. High-Temperature Drop Calorimetry—Enthalpy of Formation

**Figure 3:**
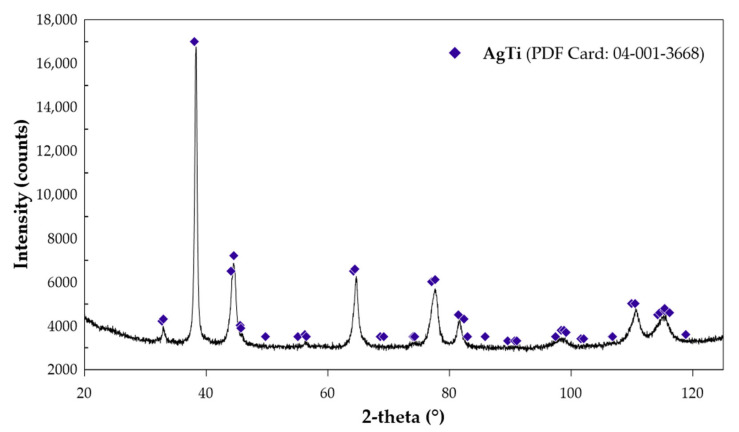
X-ray diffraction pattern of the AgTi phase after a direct calorimetric synthesis measurement.

The experimental data on the enthalpy of formation of the AgTi intermetallic phase, obtained using the direct synthesis method, are summarized in Table 6. After each measurement, the reaction crucible’s content was examined using the X-ray Diffraction (XRD) method to verify the product formed during the experiment. The diffraction pattern for the first sample is shown in Figure 3.

Based on three measurements, the average enthalpy of formation (Δ_f_*H*) for AgTi was calculated as −2.4 (±0.3) kJ/mol∙at. A summary of the literature data, supplemented by the values determined in this study, is presented in Table 7. Upon analyzing the literature data, it becomes clear that the value determined for the AgTi phase in the present work is in good agreement with the calculated values presented in [13]. In the case of the experimentally determined values reported in [20], observed difference is 0.8 kJ/mol at. However, due to the large measurement uncertainty, it remains within the range of error, and therefore our data can be considered satisfactorily consistent with the results reported in [20].

### 2.2. Theoretical Study

#### 2.2.1. Thermodynamic Optimization—CALPHAD

The thermodynamic parameters elaborated in this study are summarized in Table 8.

**Figure 4:**
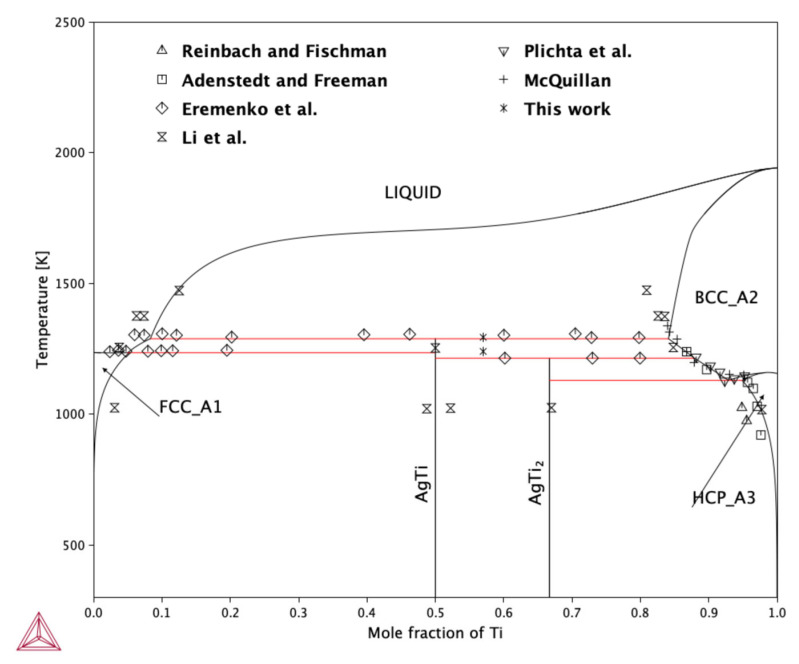
Calculated phase diagram of the Ag-Ti system, superimposed with the experimental data from Reinbach and Fischman [24], Adenstedt and Freeman [6], Eremenko et al. [9], Li et al. [13], Plichta et al. [10], and McQuillan [8].

This set of parameters enabled the calculation of the phase diagram presented in Figure 4. For the thermodynamic optimization procedure, several data sources were utilized. These include the enthalpy of mixing reported by Fitzner and Kleppa [18], as well as the new mixing enthalpy measurements obtained in this work. In addition, the activity of Ag reported by Wei et al. [19], the phase equilibria data from various literature sources [6,8,9,10,13,24], and this study, and the formation enthalpies of intermetallic compounds—both from our ab initio calculations and calorimetric measurements—were incorporated, along with the data published by Meschel and Kleppa [20].

A key challenge in the optimization process stems from the negative mixing enthalpy of the liquid phase at low Ti concentrations, which complicates obtaining a good fit while maintaining the experimentally reported phase equilibria between the liquid and BCC-Ti phases. A closer analysis of the available phase equilibrium data suggests that the actual shape of the liquidus and solidus lines in the binary region between liquid and β-Ti might differ slightly from the one commonly reported. Most experimental data for this region were obtained using diffusion couple techniques, which may not yield true equilibrium results if the annealing time is insufficient. For instance, the results reported by Li et al. [13] are questionable due to the short equilibration time of only 24 h at 1473 K. Such a short duration is likely insufficient to achieve equilibrium at this temperature, implying that the reported compositions of the coexisting liquid and β-Ti phases should be interpreted with caution.

It is also noteworthy that no experimental determinations of the liquidus line exist at temperatures above 1473 K. As a result, the shape of the high temperature liquidus line, initially proposed by Murray and Bhansali [11] and subsequently reproduced in later studies, should be regarded as a theoretical extrapolation rather than an experimentally validated result. Furthermore, Nagase et al. [16] suggested the existence of a miscibility gap in the liquid phase. However, their study did not provide sufficient information to determine the location or temperature of the critical point. Interestingly, during the present thermodynamic optimization, the computational algorithm predicted a miscibility gap in the liquid phase. Nevertheless, due to the absence of experimental confirmation, this feature was excluded from the final thermodynamic description.

As shown in Figure 4, the incorporation of a negative mixing enthalpy for the liquid phase results in a noticeable shift in the liquidus line toward the Ti-rich side at relatively low temperatures. However, at higher temperatures, the calculated liquidus aligns well with the experimental data reported by Li et al. [13]. A similar trend is observed for the solidus line of the β-Ti phase, which in the present calculation shows a lower solubility of Ag compared to that reported by Li et al. Although Li et al.’s [13] data suggest higher silver solubility in β-Ti, their short equilibration time—only 24 h at 1473 K—raises questions about the reliability of their results, as previously discussed. In contrast, the calculated solidus line in this region shows good agreement with the data reported by McQuillan [8], indicating that the present thermodynamic description more accurately reflects the equilibrium conditions. Furthermore, the solvus line between β-Ti and the AgTi_2_ intermetallic phase shows excellent agreement with the experimental observations provided by McQuillan [8], Eremenko et al. [9], and Plichta et al. [10].

The invariant reactions predicted by the model also correspond well with experimental data, particularly those reported by Eremenko et al. [9], as well as the results obtained in this study. One minor discrepancy is observed in the peritectoid reaction in which AgTi and β-Ti form AgTi_2_. The present calculation yields a reaction temperature of 1213 K, consistent with Eremenko et al.’s [9] data, while the experimental determination in this study indicates a slightly higher temperature of 1229 K. Additionally, the solubility of Ti in the (Ag) phase, as predicted by the model, is slightly lower than that reported by Li et al. [13]. This may again be attributed to differences in experimental conditions and equilibration times, highlighting the importance of careful interpretation of diffusion couple data in thermodynamic assessments.

#### 2.2.2. Enthalpy of Formation Calculation

The formation enthalpies of the intermetallic compounds AgTi and AgTi_2_ show good agreement with both theoretical predictions and experimental measurements. Ab initio calculations yielded formation enthalpies of −5977 J/mol at. for AgTi and −5052 J/mol at. for AgTi_2_. These values are referenced to pure silver in the FCC_A1 structure and titanium in the HCP_A3 structure. In this work, the experimentally determined formation enthalpy of the AgTi phase was found to be −19,500 J/mol at. This value was recalculated to 298 K and referenced to solid Ag (FCC_A1) and solid Ti (HCP_A3), ensuring consistency with standard thermodynamic conventions. The formation enthalpies derived from the CALPHAD optimization are also in reasonable agreement with both the experimental and theoretical values. For the AgTi phase, the assessed enthalpy of formation is −6076 J/mol at. at 0 K and −17,048 J/mol at. at 298 K. These results fall within an acceptable range, supporting the validity of the thermodynamic description used in the optimization. For the AgTi_2_ phase, the CALPHAD method yields a formation enthalpy of −4633 J/mol at., which is slightly less negative than the value obtained from ab initio calculations. However, this difference is considered acceptable, as the CALPHAD approach is designed to determine a set of Gibbs energies that best fit a wide range of experimental and theoretical data. Therefore, minor discrepancies between the CALPHAD assessments and the ab initio results are expected and do not undermine the overall reliability of the model.

#### 2.2.3. Enthalpy of Mixing Calculations

**Figure 5:**
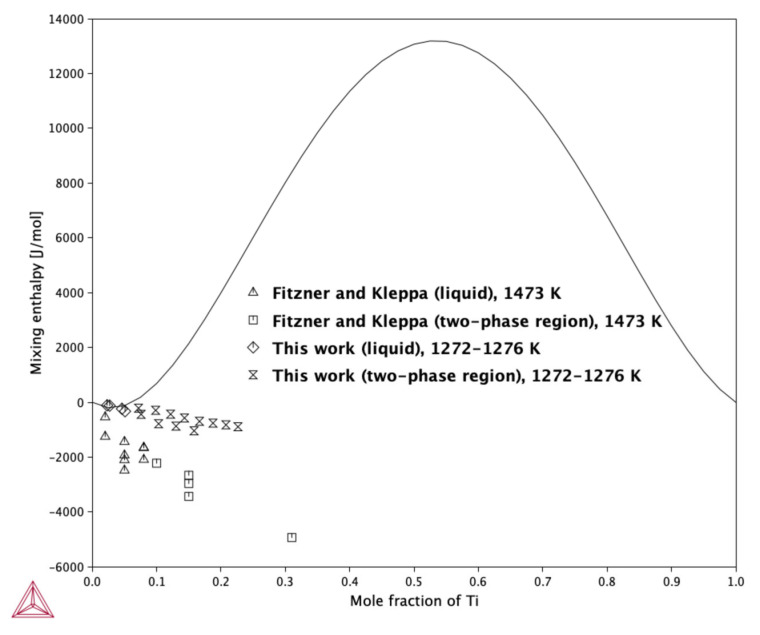
Calculated mixing enthalpy of the liquid phase at 1273 K, together with the experimental determination determined in this work and reported by Fitzner and Kleppa [18]. Reference states: Ag—liquid, Ti—liquid.

Figure 5 shows the calculated mixing enthalpy of the liquid phase at 1273 K, superimposed with the experimental determinations given in this work as well as by Fitzner and Kleppa.

Figure 5 clearly illustrates a significant difference between the mixing enthalpy reported by Fitzner and Kleppa [18] and the values determined in the present work. It is also evident that the enthalpy of mixing measured in the two-phase region follows a nearly linear trend, which is typically an indication of a reliable and accurate experimental measurement. The CALPHAD calculations performed in this study show good agreement with the new experimental data presented here but exhibit a noticeable discrepancy when compared to the values reported by Fitzner and Kleppa [18]. As discussed in our previous work, forcing the thermodynamic model to conform to the enthalpy of mixing data from Fitzner and Kleppa [18], along with the liquidus line proposed by McQuillan [6], results in the appearance of a miscibility gap in the liquid phase. However, since there is currently no experimental evidence supporting phase separation in the liquid, it would be inappropriate to propose a change to the established phase diagram morphology based solely on theoretical fitting. As shown in Table 8, the mixing enthalpy in this study has been modeled as temperature independent. An attempt was made to introduce a temperature dependence into the mixing enthalpy function, but this did not result in improved agreement with either the data of Fitzner and Kleppa [18] or the shape of the liquidus line. Therefore, a temperature-independent formulation was retained as the best compromise between experimental consistency and thermodynamic coherence.

#### 2.2.4. Silver Activity in the Liquid Phase Calculation

**Figure 6:**
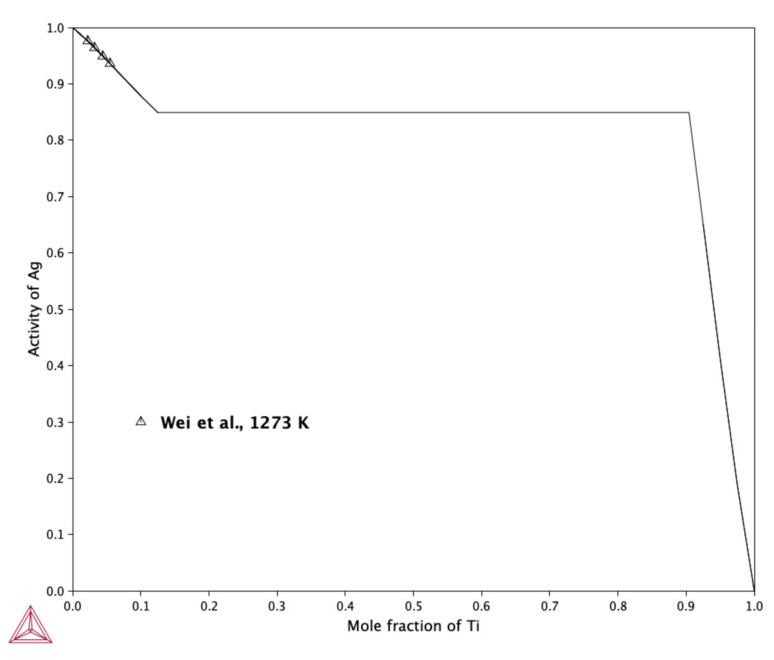
Calculated activity of Ag in the liquid phase at 1273 K, together with experimental determination by Wei et al. [19]. Reference states: Ag—liquid, Ti—liquid.

Figure 6 presents the calculated activity of silver in the liquid phase at 1273 K, referenced to pure liquid silver and liquid titanium.

The results show good agreement with the experimental measurements reported by Wei et al. [19], indicating that the thermodynamic model accurately captures the behavior of the Ag-Ti liquid solution at this temperature. It is also noteworthy that the shape of the activity curve suggests a tendency toward phase separation in the liquid phase at 1273 K. This observation aligns with the findings of Nagase et al. [16], proposed the existence of a miscibility gap in the Ag-Ti liquid system. However, due to the presence of a stable binary phase region for compositions with *x*_Ti_ > 0.1 at 1273 K, the miscibility gap cannot be observed in the equilibrium phase diagram shown in Figure 4. This limitation highlights the importance of considering both the thermodynamic predictions and the phase equilibria constraints when interpreting the activity data. Although the silver activity indicates a tendency of liquid Ag-Ti solutions to develop an immiscibility region, this region does not appear because it is determined by the change in the free energy of the solution, which has no local extremes at this temperature.

## 3. Materials and Methods

The results presented and discussed in this paper were obtained according to a methodology divided into two main parts: an **experimental study** on selected thermodynamic properties and a **theoretical study** that covers the CALPHAD (CALculation of PHAse Diagrams) and ab initio (first principles) calculations.

### 3.1. Experimental Study

#### 3.1.1. Sample Preparation

**Figure 7:**
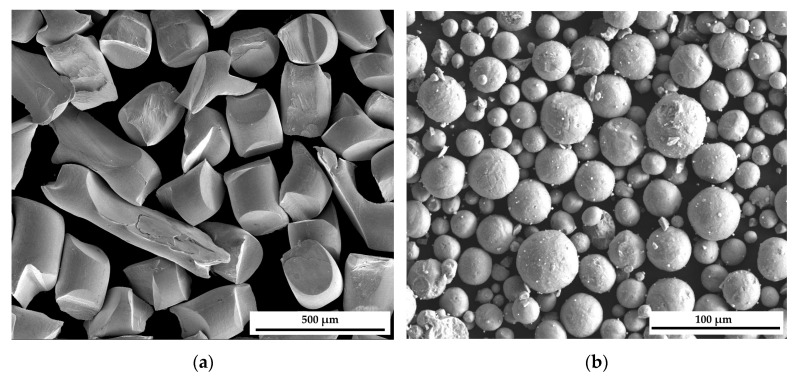
SEM/SE images of starting powder: (**a**) Silver, (**b**) TiH_2_.

The alloy Ag_0.43_Ti_0.57_ used for the DSC measurements was obtained using a mechanical alloying process. As starting materials, titanium hydride (TiH_2_) and silver were used. The TiH_2_ was produced from Grade 1 titanium (Ti-Gd1) powder supplied by Carpenter (Tanner, AL, USA), consisting of spherical particles 15–45 μm in size, while the silver was obtained in the form of irregular pieces by cutting silver wire (Innovator, Gliwice, Poland, purity 99.99%) with a diameter of 0.2 mm. SEM images of both starting materials are presented in Figure 7a,b. Loading and unloading of the powders were carried out in a glove box (MBraun LabMaster, Munich, Germany) with a high-purity environment of constantly purified argon (<1 ppm O_2_ and H_2_O). Powder mixtures with 10 stainless steel balls (10 mm diameter, AISI 304) were loaded into a 20 mL vial made from the same material as the balls and then tightly capped. Subsequently, the sample was subjected to ball milling at 400 RPM for 1 h without any pauses. 

After mechanical alloying, a fraction of the resulting powder was analyzed using SEM and XRD. The remaining powder was annealed at a temperature of 900 °C for 10 h. To minimize the risk of oxidation, the annealing furnace was placed inside a Lab Master (MBraun LabMaster, Munich, Germany) glovebox, operating under a high-purity inert atmosphere. Finally, the powder was uniaxially pressed into cylindrical samples (compacts) with a diameter of 3 mm and a height in the range of 2–4 mm, which were subsequently used for DSC measurements.

The samples used for the high-temperature calorimetry measurements were made from high-purity titanium (Alfa Aesar, Thermo Scientific Kandel GmbH, Kandel, Germany) and silver (Innovator Sp. z.o.o, Gliwice, Poland) rods with a diameter of 3 mm. They were characterized by a cylindrical shape and a height ranging from 3 to 20 mm. For mixing enthalpy change measurements, the average mass of the silver calibration samples was *m*_Ag_ = 0.1243 (±0.015) g, whereas that of the dropped titanium samples was found to be *m*_Ti_ = 0.0754 (±0.009) g. For the investigation of the enthalpy of formation, the masses of all samples were calculated so that the final composition of the alloy inside the reaction crucible was consistent with the composition of the AgTi intermetallic phase. All samples were mechanically cleaned with a file before being dropped to remove any potential surface contamination.

#### 3.1.2. X-Ray Diffraction (XRD)

XRD analysis was conducted on samples obtained through mechanical alloying and during enthalpy of formation measurements. In the first case, the powder was analyzed after mechanical alloying and subsequent annealing. This analysis was conducted on an X-ray diffractometer (Ultima IV Rigaku, Tokyo, Japan) equipped with a cobalt anode lamp (CoKα λ = 1.78897 Å) under operating conditions of 40 mA, 40 kV, and a scanning speed of 1°/min. A DeteX-Ultra fast linear counter (Rigaku) was used in continuous scanning mode with 185 parallel beam geometry. The diffraction patterns were collected at room temperature in air over a 2θ angle range of 10–120°. The samples formed during the enthalpy of formation measurements were analyzed using a Panalytical Empyrean diffractometer with Cu-Kα radiation (λ = 1.54 Å). The analysis of diffraction patterns shown in Figure 1b and Figure 3 was performed with the HighScore version 4.8 (Malvern Panalytical, Malvern, UK) software connected to the PDF5+ database (ICDD, Newtown Square, PA, USA).

#### 3.1.3. Scanning Electron Microscopy (SEM), Energy Dispersive X-Ray Spectroscopy (EDS)

To determine the chemical composition and morphology of the alloy obtained through a mechanical alloying process, an analysis was conducted using a Scanning Electron Microscope (FEI Quanta 3D, Hillsboro, OR, USA) equipped with an energy dispersive X-ray spectrometer EDS (Thermo Fisher Scientific, Waltham, MA, USA). The chemical composition shown in Table 3 represents the arithmetic average of three measurements carried out using the point analysis method on individual powder particles within the sample. The chemical composition was determined by EDS (EDAX), using the Ag L (Lα, Lβ) and Ti K (Kα, Kβ) characteristic X-ray transitions. Quantification was performed using the EDAX ZAF mode in standardless mode.

#### 3.1.4. Differential Scanning Calorimetry (DSC)

Differential Scanning Calorimetry of the Ag_0.43_Ti_0.57_ sample with a mass of m*_alloy_* = 105.73 mg was conducted using a DSC 404 F1 Pegasus calorimeter (Netzsch, Selb, Germany). The temperature range of the analysis was 323–1693 K, with the heating rate of 10 K/min. During the measurement, the sample was placed in an Al_2_O_3_ crucible with a lid, and an empty Al_2_O_3_ crucible with a lid was used as a reference. The process was performed in an inert atmosphere of high-purity argon, providing a protective environment for the experiment. The obtained calorimetric curves were analyzed using the analytical software Proteus 6.1 produced by Netzsch.

#### 3.1.5. High-Temperature Drop Calorimetry—Mixing Enthalpy Change

The mixing enthalpy change for liquid Ag-Ti alloys within the concentration range *x*_Ti_ = 0.02–0.226 was measured in two series using the high-temperature drop calorimetry technique. A comprehensive description of the measurement parameters for both series can be found in Table 9.

The procedure for each experiment can be divided into three main stages. The initial stage of the process was the preparation of the measurement. In this step, the reaction crucible with a small quantity of silver bath was placed inside the calorimeter Setaram MHTC 96 line evo (Setaram Instrumentation—KEP Technologies, Caluire, France). After that, the device was purified several times by evacuation with a vacuum pump and flushed with high-purity argon (Pioniergas, Krakow, Poland). At the end of this initial stage, the calorimeter temperature was raised to the measurement temperature.

Subsequently, the calibration process was performed, and the calibration coefficient *K* was determined based on six thermal effects from the silver samples dropped into the reaction crucible. The calculation of *K* was performed using Equation (1).

(1) $$K=\frac{{\Delta H}_{\mathrm{Ag}}^{T_{D}\to T_{M}}\cdot n_{\mathrm{Ag}}}{{\Delta H}_{\mathrm{Calibration}}}$$

where ${\Delta H}_{\mathrm{Ag}}^{T_{D}\to T_{M}}$ is the molar enthalpy difference of silver between room (*T*_D_) and measurement temperature (*T*_M_) calculated according to the relations in [25] and ${\Delta H}_{\mathrm{Calibration}}$ represents the voltage signal generated by the heat increment from the dropped calibration sample.

The final step of the measurement procedure involved determining the mixing enthalpy change based on the heat effects caused by the heat increment which comes from each dropped titanium sample. The Calisto software v. 1.39 (Setaram Instrumentation—KEP Technologies, Caluire, France) was used to analyze the thermal effects. Furthermore, the partial molar enthalpy for titanium was determined based on the observed thermal effects. The final values of the thermodynamic properties determined in this experiment were calculated using Equations (2)–(4).

(2) $$\Delta_{\mathrm{mix}}H=\frac{\sum H_{\mathrm{DISS}\text{-}\mathrm{Ti}}}{n_{\mathrm{Ag}}+\sum n_{\mathrm{Ti}}}$$

(3) $$H_{\mathrm{DISS}\text{-}\mathrm{Ti}}=({\Delta H}_{\mathrm{Signal}}\cdot K)-({\Delta H}_{\mathrm{Ti}}^{T_{D}\to T_{M}}\cdot n_{\mathrm{Ti}})$$

(4) $${∆H}_{\mathrm{Ti}}=\frac{H_{\mathrm{DISS}-\mathrm{Ti}}}{n_{\mathrm{Ti}}}$$

where $H_{\mathrm{DISS}\text{-}\mathrm{Ti}}\;$ is the enthalpy of dissolution of pure titanium, ${\Delta H}_{\mathrm{Signal}}$ is the voltage signal generated in μV·s caused by the heat increment that comes from each dropped sample, *n*_Ag_ and *n*_Ti_ stand for the number of moles of silver and titanium, while ${∆H}_{\mathrm{Ti}}$ stands for the partial molar enthalpy.

**Table 9:** Detailed measurement parameters for series A and B.

	Series A	Series B
Crucible material	ZrO_2_ + Y_2_O_3_	
Calibrant material	Silver	
Protective atmosphere	Argon at pressure *p* = 0.1 MPa	
Starting amount of silver—***n*_Ag_** [mol]	*n*_Ag_ = 0.0473	*n*_Ag_ = 0.0557
Calibration constant —***K*** [kJ∙μVs]	*K* = 8.376 × 10^−6^	*K* = 8.340 × 10^−6^
Temperature —***T*** [K]	*T*_D_*=* 298, *T*_M_ *=* 1401	*T*_D_*=* 298, *T*_M_ *=* 1400
$\mathrm{Enthalpy}\;\mathrm{Change}\;\mathrm{of}\;\mathrm{pure}\;\mathrm{elements}—{\Delta H}_{i}^{T_{D}\to T_{M}}$ [kJ/mol]	${\Delta H}_{Ag}^{T_{D}\to T_{M}}\;$= 43.3110 ${\Delta H}_{Ti}^{T_{D}\to T_{M}}\;$= 46.8315	${\Delta H}_{Ag}^{T_{D}\to T_{M}}\;$= 43.2775 ${\Delta H}_{Ti}^{T_{D}\to T_{M}}\;$= 46.7941
Standard uncertainties of the number of moles —***u*(*n*_i_)** [mol]	*u*(*n*_Ti_) = 2.09 × 10^−6^, *u*(*n*_Ag_) = 9.27 × 10^−7^	
Standard uncertainties of temperatures —***u*(*T*)** [K]	*u*(*T*_D_) = 1, *u*(*T*_M_) = 1	
Standard uncertainties of argon pressure —***u*(*p*)** [kPa]	*u*(*p*) = 10	
Standard uncertainties calibration constant —***u*(*K*)** [kJ∙μVs]	*u*(*K*) = 3.27 × 10^−7^	*u*(*K*) = 5.26 × 10^−8^

#### 3.1.6. High-Temperature Drop Calorimetry—Enthalpy of Formation

To determine the enthalpy of formation of the AgTi intermetallic phase, the high-temperature direct synthesis calorimetry method was used. This method is based on measurements of the energy effect of the reaction of the components (Ag, Ti) at a fixed temperature.

The experiment was conducted in stages. The initial steps (preparation and calibration) were analogous to the measurement of the mixing enthalpy change described in Section 3.1.5. Then, a sample of Ag, prepared as described in Section 3.1.1, was dropped into the reaction crucible with the appropriate amount of Ti placed in the calorimeter. Thus, in the reaction crucible, the synthesis of the AgTi compound took place. The reaction is described by the following thermochemical reaction:

(5) $$X_{Ag}{Ag}_{\left(T_{D}\right)}+X_{Ti}{Ti}_{\left(T_{M}\right)}\to {Ag}_{X_{Ag}}{Ti}_{X_{Ti\;(T_{M})}}+{∆H}^{ef}$$

where *T*_D_ designates the temperature of the components before the introduction into the reaction crucible (room temperature), *T*_M_ is the temperature at which the reaction occurs (measurement temperature), and Δ*H*^ef^ is the reaction heat effect measured by the calorimeter.

In this case, the enthalpy of formation Δ_f_*H* at temperature *T*_M_ is the difference between the measured heat effect of the reaction and the heat required to raise the components from temperature *T*_D_ to temperature *T*_M_ and is expressed according to Equation (6).

(6) $${∆}_{f}H={∆H}^{ef}-{(X}_{Ag\;}{\Delta H}_{Ag}^{T_{D}\to T_{M}}+X_{Ti\;}{\Delta H}_{Ti}^{T_{M}\to T_{M}})$$

where *X*_Ag,_ *X*_Ti_ are the molar fractions of the reacted components, and for the presented result $X_{Ti\;}{\Delta H}_{Ti}^{T_{M}\to T_{M}}\;$= 0.

The duration of a single measurement was approximately 60 min and continued until a constant baseline was observed. The experiment described above was carried out three times using a Setaram MHTC 96 line evo calorimeter (Setaram Instrumentation—KEP technologies, Caluire, France). As with the mixing enthalpy change measurements, the Calisto software v. 1.39 (Setaram Instrumentation—KEP technologies, Caluire, France) was used to analyze the thermal effects. The measurement parameters for each experiment are summarized in Table 10.

### 3.2. Theoretical Investigation

Two computational approaches were employed in the theoretical part of the investigation: the CALPHAD (CALculation of PHAse Diagrams) method and ab initio (first principles) calculations. Both methods were thoroughly described in our previous work on the Ag-Ti system [14], where their implementation and relevance to phase diagram modeling were discussed extensively. These approaches enable a robust and comprehensive thermodynamic description of multicomponent systems by combining experimental data with theoretical calculations. The primary distinction between the current and previous studies lies in the treatment of the mixing enthalpy of the liquid phase. In the earlier investigation, the enthalpy of mixing data reported by Fitzner and Kleppa [18] was excluded, based on substantial theoretical and experimental evidence suggesting a positive mixing enthalpy across the entire composition range of the liquid phase. This interpretation led to the adoption of a model that assumed repulsive Ag-Ti interactions in the liquid state, as discussed in detail in [14].

However, the new experimental results presented in this paper reveal that the enthalpy of mixing for the liquid phase is, in fact, negative at low Ti concentrations, indicating attractive Ag-Ti interactions in this compositional range. This discrepancy with previous assumptions necessitated a revision of the thermodynamic description of the liquid phase to accommodate the observed behavior. As a result, adjustments were also made to the parameters governing the solid phases to preserve consistency and thermodynamic coherence across the entire system. Despite these necessary modifications, the fundamental computational framework—including all models, equations, and calculation procedures—remains unchanged from our previous work on the Ag-Ti system [14], ensuring continuity and comparability between the two studies.

## 4. Conclusions

To summarize, this work presents the outcomes of calorimetric measurements together with the results of thermodynamic calculations for selected alloys of the Ag-Ti system. The main conclusion is the fact that there is a need for further experimental investigation into the Ag-Ti system, especially concerning the liquidus line at temperatures above 1473 K, and the enthalpy of formation for the AgTi_2_ intermetallic phase. This experimental information may prove valuable for future thermodynamic assessments of a multicomponent system comprising the Ag-Ti binary system. Based on the presented findings from the individual parts of the work, the following conclusions can be drawn:

- Concerning the results of the mixing enthalpy measurements of alloys in the range of *x*_Ti_ = 0.02–0.226, the integral molar mixing enthalpy change and partial molar enthalpy of Ti were determined based on two measurement series. The results showed negative values across the entire investigated concentration range.
- Regarding the enthalpy of formation values for the AgTi intermetallic phase ascertained calorimetrically via the direct synthesis method, the mean value was −2.4 (±0.3) kJ/mol∙at. The results reported in this study are consistent with the values calculated in [11] and fall within the range of the experimental data reported in [20]. However, the theoretically determined enthalpy of formation shows more negative values than those obtained by experiment.
- The formation enthalpies of the intermetallic compounds AgTi and AgTi_2_ show good agreement with both theoretical predictions and experimental measurements.
- The results of calculated silver activity in liquid Ti at 1273 K show good agreement with the experimental measurements reported by Wei et al. [17], indicating that the thermodynamic model accurately captures the behavior of the Ag-Ti liquid solution at this temperature.

## Figures and Tables

**Table 1:** Crystal structure data—Ag-Ti [11].

Phase	Pearson Symbol	Strukturbericht Designation	Space Group	Prototype
(αTi)	hP2	A3	*P6_3_/mmc*	Mg
(βTi)	cI2	A2	*Im3m*	W
AgTi_2_	tI6	C11	*I4/mmm*	MoSi_2_
AgTi	tP4	B11	*P4/mmm*	_γ_CuTi
(Ag)	cF4	A1	*Fm3m*	Cu

**Table 2:** Invariant reaction in the Ag-Ti system.

Reaction	Composition *x*_Ti_			Temperature [K]	Method	Reference
	**I**	**II**	**III**			
L + (βTi) →AgTi	0.083	0.841	0.500	1288	CALPHAD	This work
	—	—	0.500	1273	Melting of AgTi	[17]
	0.163	—	0.500	1311	Metallography	[6]
	0.150	0.900	0.500	1290–1303	Metallography	[8]
	—	0.940	0.500	1293	Thermal analysis	[9]
	0.058	0.845	0.500	1294.8	CALPHAD	[14]
	0.059	0.835	0.500	1289	CALPHAD	[13]
	0.085	0.836	0.500	1297	CALPHAD	[12]
AgTi + (βTi) → AgTi_2_	0.500	0.879	0.667	1213	CALPHAD	This work
	0.500	0.883	0.667	1213	Microprobe, metallography	[10]
	0.500	0.900	0.667	1173	Metallography	[6]
	0.500	0.880	0.667	1203	Metallography	[8]
	0.500	—	0.667	1218	Thermal analysis	[9]
	0.500	0.889	0.667	1212	CALPHAD	[14]
	0.500	0.887	0.667	1206	CALPHAD	[13]
	0.500	0.885	0.667	1216	CALPHAD	[12]
(βTi) → (αTi) + AgTi_2_	0.928	0.948	0.667	1129	CALPHAD	This work
	0.924	0.953	0.667	1128	Assessed	[11]
	0.934	0.947	0.667	1126	CALPHAD	[14]
	0.924	0.931	0.667	1129	CALPHAD	[13]
	0.933	0.958	0.667	1126	CALPHAD	[12]
L → (Ag) + AgTi	0.049	0.051	0.500	1234	CALPHAD	This work
	0.050	0.050	0.500	1232	Assessed	[11]
	0.061	0.057	0.500	1232	CALPHAD	[12]
L + AgTi → (Ag)	0.039	0.500	0.043	1235	CALPHAD	[14]
	0.042	0.500	0.047	1237	CALPHAD	[13]

**Table 3:** Average chemical composition of the Ag_0.43_Ti_0.57_ alloy after annealing—based on three EDS measurements.

Element	Chemical Composition	
	**[atomic%]**	**[mass%]**
Ag	42.9 (±0.6)	62.8 (±0.6)
Ti	57.1 (±0.6)	37.2 (±0.6)

The XRD analysis revealed the presence of two phases in the material, namely AgTi and Ti.

**Table 4:** Enthalpies of mixing obtained for the liquid Ag-Ti alloys—series A.

Number of Dropped Moles	Heat Effect	Drop Enthalpy	Mole Fraction	Integral Molar Mixing Enthalpy	Partial Molar Enthalpy	Standard Uncertainties
$n_{Ti}$	${\Delta H}_{Signal}\cdot K$	$H_{DISS-Ti}$	$x_{Ti}$	$\Delta_{mix}H$	${\Delta \bar{H}}_{Ti}$	$u(\Delta_{mix}H)$
**[mol]**	**[kJ]**	**[kJ/mol]**		**[kJ/mol]**	**[kJ/mol]**	**[kJ/mol]**
0.00131	0.05544	−0.006	0.0270	−0.124	−4.6	0.045
0.00122	0.04704	−0.010	0.0509	−0.327	−8.4	0.081
0.00135	0.05697	−0.006	0.0759	−0.439	−4.6	0.125
0.00155	0.05379	−0.019	0.1031	−0.783	−12.1	0.165
0.00164	0.07057	−0.006	0.1301	−0.870	−3.7	0.215
0.00181	0.07369	−0.011	0.1582	−1.040	−6.1	0.266

**Table 5:** Enthalpies of mixing obtained for the liquid Ag-Ti alloys—series B.

Number of Dropped Moles	Heat Effect	Drop Enthalpy	Mole Fraction	Integral Molar Mixing Enthalpy	Partial Molar Enthalpy	Standard Uncertainties
$n_{Ti}$	${\Delta H}_{Signal}\cdot K$	$H_{DISS-Ti}$	$x_{Ti}$	$\Delta_{mix}H$	${\Delta \bar{H}}_{Ti}$	$u(\Delta_{mix}H)$
**[mol]**	**[kJ]**	**[kJ/mol]**		**[kJ/mol]**	**[kJ/mol]**	**[kJ/mol]**
0.00131	0.05499	−0.006	0.0230	−0.112	−4.9	0.006
0.00138	0.05716	−0.007	0.0461	−0.237	−5.4	0.013
0.00162	0.07693	0.001	0.0719	−0.215	0.6	0.022
0.00177	0.07768	−0.005	0.0985	−0.293	−2.9	0.030
0.00164	0.06712	−0.009	0.1217	−0.434	−5.8	0.037
0.00160	0.06512	−0.010	0.1433	−0.572	−6.0	0.044
0.00182	0.07586	−0.009	0.1666	−0.695	−5.1	0.052
0.00173	0.07542	−0.006	0.1876	−0.759	−3.2	0.059
0.00174	0.07556	−0.006	0.2077	−0.823	−3.3	0.066
0.00170	0.07299	−0.006	0.2264	−0.892	−3.8	0.073

**Table 6:** Heat effects Δ*H*^ef^ and formation enthalpies Δ_f_*H* of the AgTi intermetallic phase. Reference state: liquid Ag and solid Ti at temperature *T_M_*.

No.	Temperature	Enthalpy Change	Heat Effect	Enthalpy of Formation	Phases in the Alloy According to XRD ^1^
		${\Delta H}_{i}^{T_{D}\to T_{M}}$	Δ*H*^ef^	Δ_f_*H*	
	**[K]**	**[kJ/mol]**	**[kJ/mol∙at]**	**[kJ/mol∙at]**	
1.	*T*_D_ = 298 *T*_M_ = 1276	Δ*H*_Ag_ = 39.1270 Δ*H*_Ti_ *=* 28.4007	17.38	−2.18	#PDF 04-001-3668—AgTi
2.	T_D_ = 298 *T*_M_ = 1272	Δ*H*_Ag_ = 38.9931 Δ*H*_Ti_ *=* 28.2837	16.71	−2.78	#PDF 04-004-3668—AgTi #PDF 04-004-9042—Ti
3.	*T*_D_ = 299 *T*_M_ = 1272	Δ*H*_Ag_ = 39.9677 Δ*H*_Ti_ *=* 28.2585	17.23	−2.25	#PDF 04-004-3668—AgTi #PDF 04-002-2539—Ti

^1^ XRD results at room temperature, after the calorimetric measurements.

**Table 7:** Enthalpy of formation of intermetallic phases—comparison.

Enthalpy of Formation ^1^		Method	Reference
**AgTi**	**AgTi_2_**		
−2.4 ^2^ (±0.3)	—	Experimental—high-temperature direct synthesis calorimetry	This work
−1.6 (±2.4)	−2.3 (±1.1)	Experimental—high-temperature direct synthesis calorimetry	[20]
−5.9	−4.6	Calculated—CALPHAD	This work
−6.0	−5.0	Calculated—ab initio (0 K)	This work
−2.37	−1.83	Calculated—Miedema Model	[23]
−2	−2	Calculated—CALPHAD	[13]

^1^ Values in kJ/mol at. ^2^ Average value based on the experimental results from Table 6.

**Table 8:** Thermodynamic parameters.

Phase	Function [J/mol at.]
Liquid	LAg,TiLiquid 0 = 52,281.3−16.4731·T
	LAg,TiLiquid 1=−14,585.1+9.2226·T
	LAg,TiLiquid 2=−51,550.4+29.4548·T
FCC_A1 (Ag)	LAg,TiFCC_A1 0=11,406.8−18.7751·T
	LAg,TiFCC_A1 1=−22,436.4+0.0074·T
AgTi	GAg:TiAgTi 0=−3863.8−315.8260·T+44.2415·T·lnT +0.5·GHSERAG+0.5·GHSERTI
AgTi_2_	GAg:TiAgTi2 0=−3195.9−205.2664·T+28.7453·T·lnT +0.333·GHSERAG+0.667·GHSERTI
HCP_A3 (αTi)	LAg,TiHCP_A3 0=33,643.3−6.0697·T
	LAg,TiHCP_A3 1=67,200.0−39.4870·T
BCC_A2 (βTi)	LAg,TiBCC_A2 0=90,364.2−10.7659·T
	LAg,TiBCC_A2 1=297,727.8−110.6601·T
	LAg,TiBCC_A2 2=198,898.4−87.5584·T

*GHSERAG*, *GHSERTI*—Gibbs energies of Ag and Ti in their SER (Standard Element Reference state).

**Table 10:** Detailed measurement parameters for the enthalpy of formation.

	No. 1	No. 2	No. 3
Crucible material	ZrO_2_ + Y_2_O_3_		
Calibrant material	Titanium		
Protective atmosphere	Argon at pressure *p* = 0.1 MPa		
Calibration constant —***K*** [kJ∙μVs]	*K* = 7.055 × 10^−6^	*K* = 7.024 × 10^−6^	*K* = 7.340 × 10^−6^
Temperatures —***T*** [K]	*T*_D_*=* 298, *T*_M_ *=* 1276	*T*_D_*=* 298, *T*_M_ *=* 1272	*T*_D_*=* 299, *T*_M_ *=* 1272
$\mathrm{Enthalpy}\;\mathrm{Change}\;\mathrm{of}\;\mathrm{pure}\;\mathrm{elements}\;—{\Delta H}_{i}^{T_{D}\to T_{M}}\;[\mathrm{kJ}/\mathrm{mol}]$	${\Delta H}_{Ag}^{T_{D}\to T_{M}}\;$= 39.1270 ${\Delta H}_{Ti}^{T_{D}\to T_{M}}\;$= 28.4007	${\Delta H}_{Ag}^{T_{D}\to T_{M}}\;$= 38.9931 ${\Delta H}_{Ti}^{T_{D}\to T_{M}}\;$= 28.2837	${\Delta H}_{Ag}^{T_{D}\to T_{M}}\;$= 38.9677 ${\Delta H}_{Ti}^{T_{D}\to T_{M}}\;$= 28.2585
Standard uncertainties of the number of moles —***u*(*n*_i_)** [mol]	*u*(*n*_Ti_) = 2.09 × 10^−6^, *u*(*n*_Ag_) = 9.27 × 10^−7^		
Standard uncertainties of temperatures —***u*(*T*)** [K]	*u*(*T*_D_) = 1, *u*(*T*_M_) = 1		
Standard uncertainties of argon pressure —***u*(*p*)** [kPa]	*u*(*p*) = 10		
Standard uncertainties of the calibration constant —***u*(*K*)** [kJ∙μVs]	*u*(*K*) = 1.47 × 10^−7^	*u*(*K*) = 1.38 × 10^−8^	*u*(*K*) = 2.47 × 10^−7^

## Data Availability

Raw data is available upon request.
